# Technical Principles and Clinical Applications of Electrical Impedance Tomography in Pulmonary Monitoring

**DOI:** 10.3390/s24144539

**Published:** 2024-07-13

**Authors:** Ziqiang Cui, Xinyan Liu, Hantao Qu, Huaxiang Wang

**Affiliations:** School of Electrical and Information Engineering, Tianjin University, Tianjin 300072, China; liuxy_168@tju.edu.cn (X.L.); hantaoqu@tju.edu.cn (H.Q.); hxwang@tju.edu.cn (H.W.)

**Keywords:** pulmonary monitoring, electrical impedance tomography, ventilation/perfusion, image reconstruction, instrumentation

## Abstract

Pulmonary monitoring is crucial for the diagnosis and management of respiratory conditions, especially after the epidemic of coronavirus disease. Electrical impedance tomography (EIT) is an alternative non-radioactive tomographic imaging tool for monitoring pulmonary conditions. This review proffers the current EIT technical principles and applications on pulmonary monitoring, which gives a comprehensive summary of EIT applied on the chest and encourages its extensive usage to clinical physicians. The technical principles involving EIT instrumentations and image reconstruction algorithms are explained in detail, and the conditional selection is recommended based on clinical application scenarios. For applications, specifically, the monitoring of ventilation/perfusion (V/Q) is one of the most developed EIT applications. The matching correlation of V/Q could indicate many pulmonary diseases, e.g., the acute respiratory distress syndrome, pneumothorax, pulmonary embolism, and pulmonary edema. Several recently emerging applications like lung transplantation are also briefly introduced as supplementary applications that have potential and are about to be developed in the future. In addition, the limitations, disadvantages, and developing trends of EIT are discussed, indicating that EIT will still be in a long-term development stage before large-scale clinical applications.

## 1. Introduction

Medical imaging techniques have undergone rapid development in recent decades. The X-ray computed tomography (CT), magnetic resonance imaging (MRI) and positron emission tomography techniques are the most widely used medical imaging modalities. However, these imaging modalities cannot be continuously performed on patients, due to the radiation, cost, and long and tedious preparation steps. The medical electrical impedance tomography (EIT) technique is a non-radioactive imaging modality, which has been frequently discussed and investigated in pulmonary diagnosis and treatment. Due to the advantages of no-radiation, hardware simplicity, and low operation cost, EIT could be an ideal functional complement to its radioactive counterparts [1,2].

Standard EIT imaging relies on the differences in conductivity and performing properties of materials/tissues to generate image contrast [3]. Recent decades have seen the development of several new functional EIT techniques that have expanded the use of these properties to start to probe the underlying tissue impedance changes. By performing EIT imaging, the impedances and variations in tissues could be directly observed in the tomographic images. The image contrast will be more significant if the greater impedance changes exist in the tissues. This is especially true in monitoring ventilation process, in which the air volume variations in lung always results in significant changes in chest impedance. In addition, once the tissue lesion occurs, it will be reflected in the EIT images, even at the early stage of pulmonary disease. Therefore, the long-time and continuous imaging capability of EIT makes it a good candidate for performing bedside monitoring of pulmonary diseases.

**Figure 1:**
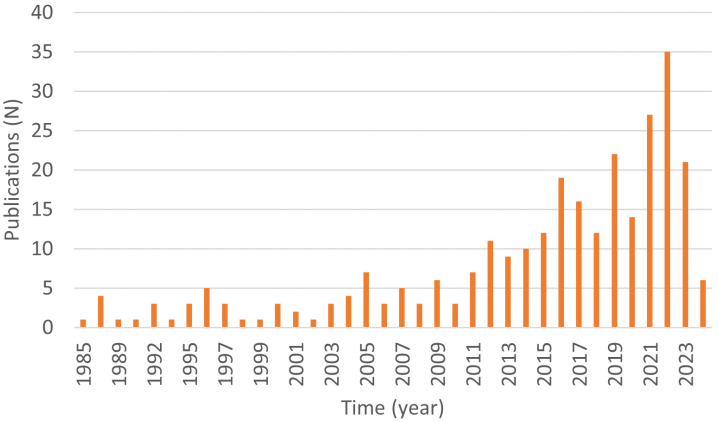
Numbers of publications on EIT applications in pulmonary monitoring. Results based on Pubmed search ((“EIT” OR “electrical impedance tomography”) AND (“pulmonary” OR “chest” OR “lung”) AND “application”).

The applications of EIT for pulmonary monitoring have seen rapid development in recent decades. The earliest EIT medical application appeared in 1978 [4] to perform the impedance imaging of the thorax. Figure 1 demonstrates the number of publications on EIT applications in pulmonary monitoring. It could be seen that the number of publications has been rising since the 21st century, and its upward trend has been faster in the past decade. Especially after the epidemic of coronavirus disease, the field experiences a rapid boom and reaches its peak in 2022. The overall trend indicates the upward trend and vigorous development of this technology.

EIT has been categorized into functional imaging modality, as opposed to the structural imaging modalities, i.e., X-ray, CT, and MRI. Usually, tomography can only provide relatively low spatial resolution as compared with its radioactive counterparts, which is considered to be related to its sensing principle. EIT relies on boundary impedance measurements to map the internal conductivity and permittivity distributions, in which it requires complicated mathematical methods.

Usually, the EIT functional images could not be intuitively understood, making it difficult to be widely accepted by clinical researchers. There still exists a long way to establish the confidences in applying EIT technique in clinical applications. Therefore, this review aims to provide a brief summary of successful EIT applications in pulmonary monitoring, and at the same time, discuss the physical limitations that caused the relatively low image resolution. At last, the development trends of the EIT technique will also be discussed.

The organization of this review is as follows. Section 2 looks at the EIT instrumentation, including the safety regulations, excitation and measurement pattern, and electrode configuration. Section 3 summarizes the EIT image reconstruction algorithms, i.e., the absolute algorithms, time-difference, frequency-difference algorithms, and the intelligent algorithms. Subsequently, Section 4 reviews clinical applications of EIT. Finally, Section 5 provides the conclusions, including the primary challenges and possible perspectives.

## 2. Instrumentation Aspect

The EIT instrumentation accounts for the real-time collection of impedance measurements. It requires the some necessary hardware to accomplish this task, including the electrode array, impedance measuring circuits, analog switch/multiplexer array, and a processor to coordinate this data collection process.

In applying EIT for clinical imaging, it is not necessary for operators to know the technical details of EIT hardware and software. However, the users should establish a clear model of EIT measuring and imaging process. In this section, we will discuss safety consideration, measuring the principle and algorithm limitations of EIT instrumentation.

### 2.1. Safety Regulations

In performing EIT measurements on the human body, safety is always the first consideration. The EIT measurement process is composed of a series of repeated and consecutive trans-impedance measurements.

Basically, the trans-impedance measurements involves applying an alternating current *I* and collecting the potential differences *U* between any different electrode pairs, or applying an alternating voltage to the body and measuring the resultant currents. The applied current constitutes the primary safety concern in EIT measurement. The safety consideration in applying electrical current on the human body has already been extensively investigated and discussed [5,6,7,8,9,10].

**Figure 2:**
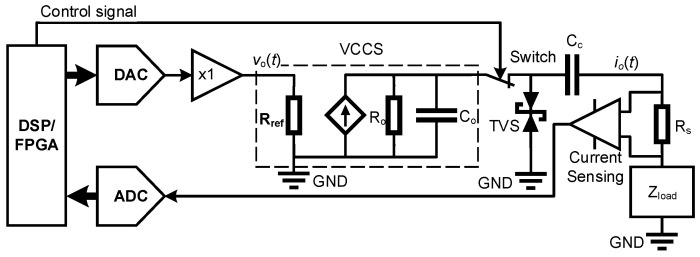
Current source in EIT system.

Generally, the voltage value does not necessarily reflect the danger criterion, i.e., a voltage below 50 V is not dangerous to the human body. The danger criterion is the current that flows through the important organs, i.e., the heart and lung for pulmonary EIT. Therefore in developing EIT system, it is usually preferred to adopt the constant current source rather than voltage source, even though the voltage source is of relatively simple structure and high output impedance. Still, the high-amplitude alternating current could be hazardous to the human body in various aspects. By applying a constant current to the human body, the total amount of current could be limited by employing proper protection circuit, as schematically demonstrated in Figure 2.

In this case, the Howland circuit has been implemented as current source. Its output current $I_{o}$ can be calculated as follows:

(1) $$I_{o}=\frac{V_{o}}{R_{ref}}$$

Generally, by applying a proper resistor $R_{ref}$, the current could be adjusted. In addition, a coupling capacitor is placed at the output of the current source in order to isolate the DC signals. It is also required to install a transient voltage suppressor (TVS) diode or other voltage-limiting components between the current source output and protection ground, which could limit the applied voltage to below the safety standard.

The literature suggests that the value of perception current ranges from 0.3 mA to 90 mA, depending on several factors. Generally, the perception current is 0.5 mA. Therefore, in developing the EIT system, it is usually required to adopt the current source rather than voltage source, even though the voltage source is of relatively simple structure and high output impedance [11,12].

By applying a constant-amplitude alternating current on the human body, the total amount of current could be physically limited, as schematically illustrated in Figure 2.

In this case, the combinational use of DSP/FPGA and analog-to-digital converter could generate a sinusoidal signal, which is buffered and converted into an alternating current by a voltage-controlled current source (VCCS). The current signal is of constant amplitude. Usually, the VCCS is implemented with the Howland circuit [13]. By applying a proper resistor $R_{ref}$, the output current could be adjusted, which can be expressed as follows:

(2) $$i_{o}\left(t\right)=k\frac{v_{o}\left(t\right)}{R_{ref}}$$

In addition, a coupling capacitor $C_{c}$ is placed at the output of the current source in order to isolate the DC signals. The current will be constantly monitored by the current sensing instrumentation amplifier. Once the current exceeded the safety value, the processor (DSP/FPGA) could switch off the circuit. In addition, it is also required to install a transient voltage suppressor (TVS) diode or other voltage-limiting components between the current source output and protection ground, which could limit the applied voltage to below the safety standard.

If the excitation current exceeded a safety level, the applied current of EIT may cause injury to the human body [14]. The high current intensity could cause rhythm disturbances in the heart, i.e., ventricular fibrillation. Generally, the ventricular fibrillation could be monitored by using ECG signals. However, its accuracy may be affected by the applied current in performing EIT measurement. Therefore, real-time monitoring funtions, i.e., ECG, should be incorporated into EIT devices to continuously detect any potential issues, and alert the operator safety risk.

In EIT, the impedance load, i.e., $Z_{load}$ in Figure 2, consists of the skin contact impedances and tissue impedance. The skin contact impedances are much more investigated than the tissue impedance, which may affect the measurement accuracy if not been properly handled. On the instrumentation aspect, the so-called tetra-polar measurement strategy has been employed, i.e., applying a constant current source through one pair of electrodes and measuring the potential differences from other electrode pairs. In this way, the effect of contact impedance could be removed from the measurements. However, it is still needed to reduce the contact impedance, i.e., to reduce the load of current source. Dry skin offers higher impedance, whereas the skin and measuring electrode immersed under conductive liquid could offer the lower resistance.

**Figure 3:**
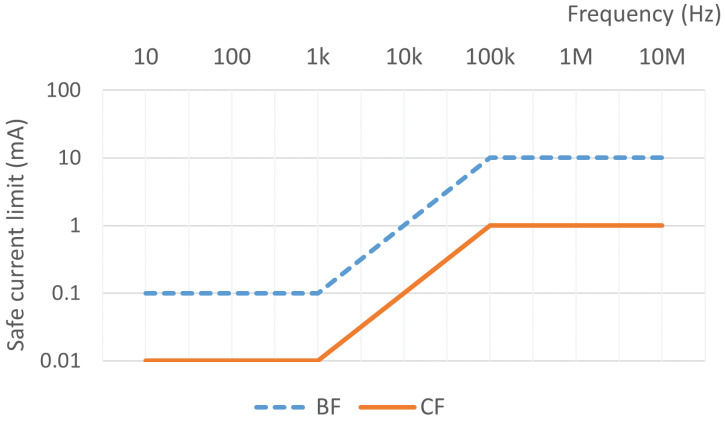
Allowable injection currents according to the BF- and CF classes of medical devices.

In the pulmonary EIT applications, there may exist two situations, i.e., the CF (cardiac floating) and BF (body floating) types. The CF-type applies to the applications wherein the devices are in direct electrical contact with the heart, e.g., dialysis equipment, monitors, and electrocardiographs. Obviously, the injection current of CF-type devices should comply with the strictest standard. The BF-type devices are typically employed in the applications wherein the conduction or long-term contact occurs with the patients, e.g., blood pressure testers, incubators, and ultrasound devices. It has been investigated that, the safe current applied for the BF and CF-type devices is illustrated in Figure 3.

Currently, most of the EIT systems apply an alternating current of 50–250 kHz frequency and 3∼10 mA amplitude [15], i.e., meeting the standard of BF-type. Considering that EIT has been frequently investigated in the medical applications, it is needed for the developers and users to identify the proper safety standard of the EIT device to be used.

Skin irritation and potential allergic reactions that are caused by the metallic electrodes are also potential hazards to the users. In this case, the use of textile material could be considered. Becher et al. conducted a pulmonary experiment with neonates and young children [16]. In the experiment, only 10% of patients incurred minor skin irritations and no one had moderate or severe adverse effects. Sophocleous et al. also found that the use of EIT for continuous chest monitoring did not cause any discomfort for preterm infants [17]. These results validate the suitability of EIT for long-time medical monitoring.

### 2.2. Excitation and Measurement Pattern

Generally, the EIT instrumentation could be viewed as a specific multi-channel impedance measurement system. In fact, some commercial impedance measurement instruments have been employed to implement the EIT system, or other electrical tomography systems [18,19,20]. In this way, it could continuously interrogate the trans-impedances between any electrode pairs on the plane of the chest [21], which is an electrically scanning process.

Basically, the impedance measurements could be achieved through the current excitation and voltage measurement, or vice versa. The excitation pattern usually refers to the type of applied signal, and the selection of current injection electrode pair, while the measurement pattern involves the type of measuring signal and the selection of measuring electrode pairs.

**Figure 4:**
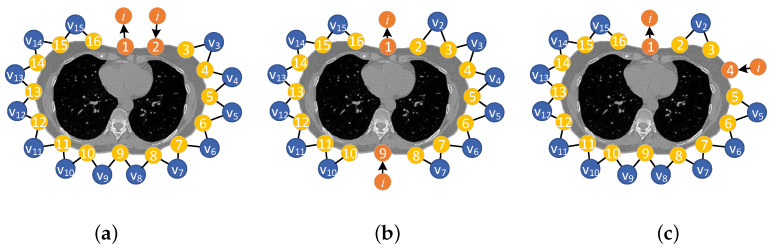
Excitation and measurement patterns of a 16-electrode EIT sensor: (**a**) adjacent, (**b**) opposing, and (**c**) two-electrode spaced electrode pairs.

The most frequently discussed pattern is the so-called adjacent pattern, which utilizes the adjacent electrode pairs for both current injection and voltage measurement [22,23], as illustrated in Figure 4a. Subsequently, all the electrode pairs are interrogated as excitation or measuring electrodes, forming a complete measurement set consisting of n×(n−3)/2 independent measurements, where *n* is the number of electrodes.

The use of opposing electrodes as current injection electrode pairs is another well-developed pattern, as illustrated in Figure 4b. Generally, in the opposing pattern, more current could be sent into the center part of the body. However, it generate less independent measurements as compared with the adjacent pattern, as can be found in Figure 4. The patterns of using the electrode pairs that are spaced out by one or more electrodes for current injection have also been discussed, as demonstrated in Figure 4c.

In most of the EIT devices, the adjacent pattern is the most widely adopted pattern, while it becomes difficult to achieve a high signal-to-noise ratio as the number of measuring electrodes increases to 32 or greater. It usually needs to inject the current through the non-adjacent electrode pairs [24] in order to pass more current into the body center, especially in the three-dimensional (3D) case. However, most 3D EIT sensor models are based on multi-layer ring electrodes. Liu et al. proposed the so-called back electrode excitation measurement (BEEM) strategy, which selects the optimal number of excitation electrodes according to the imaging effects [25]. Yang et al. proposed the use of a support vector machine (SVM) method to determine the optimal pattern, [26]. In the case of eight electrodes, it employs a 13-bit binary number to represent the permutation and combination of excitation and measurement patterns, which transforms the problem of pattern optimization into the optimal pattern recognition problem.

In most cases, the selection of excitation and measurement pattern depends on the investigated object. For example, the adjacent excitation pattern is suitable for chest imaging, while the opposing excitation pattern is frequently employed for imaging brain activities. Generally, pattern selection should focus on the information amount and/or image spatial resolution [27,28].

### 2.3. Electrode Configuration

Electrode configuration refers to the arrangement and placement of electrodes on the body surface. Typically, EIT devices utilize 8, 16, and 32 electrodes per plane [24], which are usually integrated onto an electrode belt to facilitate measurement. Most EIT systems employ wet electrodes, i.e., using conductive gels to improve the connectivity. In this case, attention should be paid to the evaporation of conductive gel between the electrode and skin [29,30].

However, the signal quality may be deteriorated for the current may pass through the gel layer rather than the chest tissue, which contributes to the current leakage. In addition, the wet electrodes may be non-adapted and uncomfortable for the patient, and cause the risk of skin injury. To solve this problem, Brabant et al. recommended the gel with low conductivity and water solubility, which could reduce the air gap between the electrode and the skin [31]. Lin et al. designed a wearable wireless EIT belt with dry electrodes, eliminating the limited applicability of wet electrodes [30]. Hu et al. developed the textile electrodes integrated in a clothing belt [29]. These studies indicate that there is a clear trend to replace the wet electrodes with dry electrodes to compensate for error.

Sophocleous et al. developed a 32-electrode textile interface which is durable, stretchable, and thoroughly clean [17]. A stable contact impedance of about 300 Ω with 5 Ω tidal variation of contact impedance was observed during the 20 min period without any distress or discomfort for the preterm infants.

Generally, the EIT electrodes are placed near the 5th intercostal space to minimize the impedance disturbances. The main problem of a too low position of the electrode belt is that the diaphragm enters the registered area and therefore a reliable assessment of the lungs is no longer possible. At the same time, if the position is too high, it may lead to false hyperinflation [32]. Zhao et al. reported that the electrode belt ought to be adjusted appropriately according to individuals [33]. The electrode configurations and placements used in other works have been listed in Table 1.

In medical applications, however, the electrode offset could not be avoided, despite the negative influence on the image reconstruction. Placing electrodes too firmly may cause discomfort to patients; therefore, it should not be considered as a good solution. At the same time, the physiological activities of patients may also cause the offset. Therefore, it may be preferred to find a method to handle the electrode offset/movement rather than to totally avoid it. Shi et al. developed a method to correct the boundary voltages affected by the offset of electrodes [34]. The mapping between the offset angle variation in boundary voltages were established, while the offset electrode and angle could be obtained. The simulation verified the feasibility in correcting the error caused by electrode offset. Soleimani et al. proposed a one-step regularized algorithm, which could directly reconstruct the electrode movements and directions [35]. It was pointed out that, once the precomputation was completed, it would take very short computational time to reconstruct the image. Lozano et al. analyzed the measurement error caused by the electrode replacement and body postural changes, and recommended to use the reference position to measure the impedances that are less sensitive to postural changes [36]. In addition, the ratio of impedance at two different frequencies could be utilized to discern the impedance changes from physiological phenomena or postural errors. However, the methods mentioned above cannot completely eliminate the influence of electrode offset. Further research is still needed to better solve the errors that caused by electrode offset/movement.

In performing EIT measurements and image reconstruction, the reference electrode is indispensable. Lin et al. has conducted a series of experiments with 30 lung-healthy volunteers, under the different conditions of reference electrodes [37]. A deviation score was calculated to quantitatively investigate the influence of the reference electrode on the image. Experimental results show that the reference electrode could lead to a great impact on the quality of the images. Therefore, it is crucial to ensure the proper function of the reference electrode prior to EIT measurement. Due to the uncertainty of the measured reference voltages, Yu et al. established a nonlinear mapping between the voltage measurements and the reference voltage, indicating the potential of dynamic estimation of the time-difference reference voltage [38].

The low risk of EIT has been verified in recent research, and it is a major task to minimize its impact on the human body, such as skin irritation and other minor injuries in the future. The basic structures of safety current and excitation/measurement pattern have not been greatly changed in recent years. However, their selection has undergone more variable situations with the recent development of EIT toward 3D, which has brought more challenges. There are a significant number of recent works struggling with the electrode offset/movement. As the team of Shi et al. did, it would be more realistic and important to develop a proper method that could compensate for the error than to firmly attach the electrodes.

By continuously improving the excitation and measurement pattern especially applied on 3D situations, the more reliable and comprehensive information would be acquired from the raw measurements, which could lead to better image reconstruction results. Similarly, the selection of electrode configuration ought to consider objective factors to achieve an optimal balance between image quality and practical feasibility.

## 3. Image Reconstruction Algorithms

The image reconstruction algorithm is another key factor that determines the overall performance of EIT imaging, including the image accuracy and rate.

### 3.1. Classification of Algorithms

The EIT image reconstruction is a nonlinear, ill-posed, and under-determined problem, primarily due to the “soft-field” characteristic of electric field and limited available measurements. In comparison, CT is a “hard-field” tomography, in which the electron travels in a straight line and will not be affected by the material distribution.

Generally, the image reconstruction algorithms could be categorized into the conventional sensitivity-based algorithms and the intelligent algorithms that utilize neural networks. The conventional algorithms aim to solve the inverse problem by approximating the real conductivity map in the sense of least squares, while the intelligent algorithms intend to obtain the tomographic images from a trained neural network.

The conventional algorithms can be further classified into the absolute and difference imaging methods, considering whether the absolute conductivity or conductivity differences are obtained with the algorithms. According to the solving procedure, the conventional algorithms could also be classified into the iterative and non-iterative algorithms. In the medical use of EIT technology, the solving procedure is usually not the primary concern, except that a good iterative algorithm may help improve the image quality. Therefore, compared with iterative and non-iterative algorithms, the absolute and difference imaging methods usually affect the reconstruction results to a greater extent for the end users, in that they actually provide totally different images.

In performing the time-difference imaging, the reference measurement should be obtained at a proper time to achieve the optimal imaging effect. However, this is usually a trivial and test process. The difference imaging algorithms could reduce time consumption, and compensate the system noise by performing the subtraction operation. According to Frerichs et al., the time-difference imaging is well suited to track the time-varying physiological phenomena, e.g., the lung ventilation and perfusion [39].

### 3.2. Difference Algorithms

The difference algorithms are the most frequently discussed algorithms, which produce tomographic images corresponding to the relative change in conductivity Δσ(x,y,z) in the region of interest (ROI). In using difference algorithms, there always exists a reference measurement $u_{r}$, i.e., to calculate the measurement differences Δu.

(3) $$\Delta u=u-u_{r}$$

According to the selection of reference measurements, the difference algorithms can be categorized into the time-difference and frequency-difference algorithms. In employing the time-difference algorithms, the measurement differences are the relative change in measurements with respect to a measurement set obtained at $t_{0}$, i.e.,

(4) $$\Delta u=u_{t}-u_{t_{0}}.$$

While in the frequency-difference algorithms, the difference is calculated from the measurements at different frequencies, i.e.,

(5) $$\Delta u=u_{f}-u_{f_{0}}.$$

Generally, the time-difference measurements are obtained at a fixed frequency, which does not make full use of the impedance spectral information. In comparison, the frequency-difference measurements could take into account of the frequency-dependent bio-impedance information.

#### 3.2.1. Time Difference

In achieving the time difference imaging, there exist a number of image reconstruction algorithms, among which regularization algorithms are the most popular algorithms. The Tikhonov regularization algorithm can smooth the reconstructed image between the heart and the lungs, and stabilize the temporal changes in the conductivity [40].

For the linear problem CX=Y, the standard form and general form of $\ell_{2}$ Tikhonov regularization are [41]

(6) $$X_{\lambda }=\arg\min\left\{{∥CX-Y∥}_{2}^{2}+\lambda {∥X∥}_{2}^{2}\right\}$$

and,

(7) $$X_{\mathrm{reg}}=\arg\min\left\{F_{0}\left(CX-Y\right)+\sum_{i=1}^{q}\lambda_{i}F_{i}\left[L_{i}\left(X-X^{*}\right)\right]\right\}$$

where ${∥\mathrm{CX}-Y∥}_{2}^{2}$ and ${∥X∥}_{2}^{2}$ are the model fit and penalty term, respectively; λ is regularization parameter; $X^{*}$ is a prior guess of the solution; *q* is the number of penalty terms; $L_{i}$ is a regularization matrix; $\lambda_{i}$ is corresponding regularization parameter and $F_{i}$ is “penalty” function. Equations (6) and (7) are the usual standard-form and general Tikhonov regularization formulation, respectively. The penalty term of regularization takes the $\ell_{2}$ norm [41], which can penalize the variations in conductivity due to the smooth assumption to stabilize the inverse solution [40]. However, the over-smoothing phenomenon of the Tikhonov algorithm is a defect, and the boundary of the heart and lungs cannot be detected exactly due to cardiac activities. The Tikhonov algorithm has usually acted as the baseline to develop new algorithms. Wang et al. proposed an innovative hybrid iterative optimization algorithm that improved the quality of Tikhonov-based reconstruction [42]. As compared with Landweber, Newton–Raphson, and classical Tikhonov, it could offer a better accuracy of image reconstruction in both the simulation and experiment cases. Shi et al. combined the Tikhonov regularization with the noise reduction algorithm and developed a joint algorithm, which could provide an accurate reconstruction [43]. Wang proposed a one-step proximal method based on non-stationary iterative Tikhonov regularization [44]. It could improve the image quality, computational rate, robustness to noise, and spatial resolution, which can achieve 3.69 relative error and 0.9809 correlation coefficient at a 3% noise level.

In addition, Sun et al. improved the Tikhonov regularization algorithm to monitor lung cancer. Various electrical conductivity measurements were performed on the normal and cancerous living tissue removed from 109 patients undergoing surgery for lung cancer [45]. The numerical simulations and experiments demonstrated the feasibility and effectiveness of the proposed algorithm.

Several works are shown in Table 2. It should be noted that the “Result and Performance” column displays the data obtained from numerical simulations.

#### 3.2.2. Frequency Difference

The conventional algorithm of frequency-difference imaging is frequency-difference algorithm (FD). Subsequently, the weighted frequency-difference (WFD) algorithm is developed, which uses the “weighted” difference of boundary voltage vectors under two excitation frequencies [46]. The equations of FD and WFD algorithms are expressed as follows:

(8) $$\begin{array}{cc} \; & A\left(x_{\omega_{2}}-x_{\omega_{1}}\right)=b_{\omega_{2}}-b_{\omega_{1}} \end{array}$$

(9) $$\begin{array}{cc} \; & A\left(\alpha x_{\omega_{2}}-x_{\omega_{1}}\right)=b_{\omega_{2}}-\alpha b_{\omega_{1}} \end{array}$$

(10) $$\begin{array}{cc} \; & \alpha =\frac{\langle b_{\omega_{2}},b_{\omega_{1}}\rangle }{\langle b_{\omega_{1}},b_{\omega_{1}}\rangle } \end{array}$$

where *A* represents the sensitivity matrix with a Jacobian form; $x_{\omega_{1}}$ and $x_{\omega_{2}}$ are the conductivity distribution vectors in the measured field at frequencies $\omega_{1}$ and $\omega_{2}$, respectively; $b_{\omega_{1}}$ and $b_{\omega_{2}}$ are the boundary voltage vectors at $\omega_{1}$ and $\omega_{2}$, respectively; $\langle .,.\rangle$ represents the inner product of two vectors. Equations (8) and (9) are the FD and WFD equations, respectively.

The FD algorithm is appropriate for the cases of homogeneous conductivity, while the WFD algorithm is appropriate for the frequency-dependent background conductivity. In addition, the WFD algorithm can eliminate the frequency dependence of background conductivity by using the weighted difference of boundary voltage vectors at two excitation frequencies.

However, the performance of WFD is not adequate if the measured field contains multiple backgrounds with different conductivities. To address this problem, Hu et al. proposed an expanded multiple weighted frequency-difference (EMWFD) algorithm to precisely image different backgrounds [46]. The analysis and comparison of three algorithms were presented from both simulation and experimental aspects, which displayed the good performance of the proposed EMWFD algorithm. However, the authors pointed out that EMFWD needed more mathematical analysis to improve the imaging quality when too many backgrounds existed in the measured field.

The advantages and drawbacks of time-difference and frequency-difference algorithms are compared in Table 3, which could be selected according to actual working conditions. It should be pointed out that the time-difference and frequency-difference algorithms are not completely opposite. The information of the frequency-difference algorithm can be used as a supplement to the time-difference measurements. The combination of time-difference and frequency-difference imaging algorithms has been investigated to achieve better results. Jiang and Soleimani introduced the capacitive coupled EIT and utilized it to investigate both time-difference and frequency-difference imaging algorithms [47]. Cao et al. proved that the integration of multi-frequency and time-difference information could considerably improve the system’s performance [48]. In Bai et al.’s research, the frequency and time difference algorithms were combined together to image the bio-material inclusions and non-conductive objects simultaneously [49]. The proposed fusion algorithm could reconstruct bio- and non-conductive targets in lung phantoms, which was expected to be helpful for the diagnosis of lung-related diseases. The works are shown in Table 4. The third column without clear parameters indicates that the work is in the stage of visually observing the reconstruction shape and edges.

### 3.3. Absolute Algorithm

The algorithm’s aim is to calculate the absolute conductivity σ(x,y,z). In contrast, the aforementioned difference algorithms solve the conductivity difference Δσ(x,y,z). Generally, the absolute algorithms are complex and time-consuming as compared with the difference algorithms. Moreover, the ill-posedness of the inverse problem and the error in the system model aggravate the difficulty in performing absolute imaging.

As a non-iterative algorithm, the D-bar algorithm addresses nonlinear problems by transferring the requirement to the time-consuming forward model calculation [50]. Martins et al. presented an introduction to the D-bar algorithm and other algorithms, in which the D-bar algorithm could achieve the best temporal resolution [51]. The D-bar algorithm could be expressed as follows:

(11) $$\mu \left(z,s\right)=1+\frac{1}{{\left(2\pi \right)}^{2}}\int_{R^{2}}\frac{t(k)}{\left(s-k\right)\bar{k}}e_{-z}\left(k\right)\bar{\mu (z,k)}dk_{1}dk_{2}.$$

where *z* is a point defined in $R^{2}$; *k* is a complex parameter; t(k) represents the scattering transformation from voltage to current density; μ(z,k) represents the solution of weak singular Fredholm integral equation.

**Figure 5:**
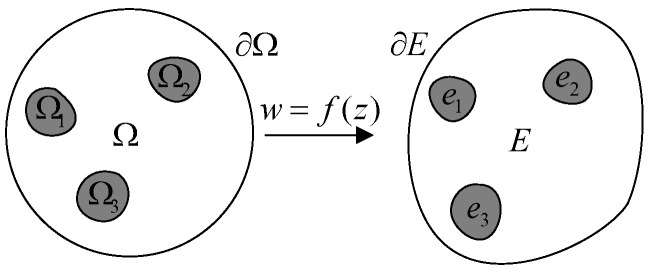
Conformal transformation from unit circle to single connected domain.

The major advantage of D-bar algorithm is the non-restriction of ROI. The measurements obtained from the circular region could be mapped to a non-circular region by conformal transformation, as schematically illustrated in Figure 5.

Here, f(z) indicates the conformal transformation from the unit circle Ω to a general continuous region *E*. Any point *w* in region *E* could be transformed by the point *z* in region Ω, while the subregions $e_{1}$, $e_{2}$, $e_{3}$ could be transformed by the subregions $\Omega_{1}$, $\Omega_{2}$, $\Omega_{3}$. Simultaneously, the value in Ω remains unchanged during the transformation. Thus, the reconstruction process could be conducted in the unit circle Ω, and transformed to the region *E* subsequently. This property makes the D-bar algorithm well suitable for monitoring the object with complex shapes, e.g., the lungs.

Hamilton made great contributions to the improvement of the D-bar algorithm [50,52,53,54,55]. Hamilton proposed the prior D-bar algorithm combining the high-confidence data, which was a noise-robust regularization algorithm [50]. The effectiveness of the proposed algorithm was verified by the experiment of chest phantom, which consisted of the heart and lungs made by agar. Subsequently, Hamilton et al. utilized the phantom model to prove that the D-bar algorithm was robust to the modeling errors, and the concomitant artifacts were similar to those that appeared in the time-difference imaging [52]. This discovery enabled the clinical application of absolute imaging which was previously considered impossible. In addition, Hamilton et al. found that the D-bar algorithm had a position-independent point spread function (PSF), suggesting that it was less sensitive to the electrode offset [55]. This might bring additional benefit to the clinical application of the D-bar algorithm. As mentioned above, the misalignment of electrodes is usually inevitable in the clinic.

### 3.4. Intelligent Algorithms

Recently, the intelligent algorithms have attracted more attention in addressing the image reconstruction of EIT. A large number of intelligent algorithms have been investigated, including the neural network (NN) algorithms, the particle swarm optimization algorithms, the genetic algorithms [56], learning-type algorithms, etc. Generally, their common feature is the capability to simulate some natural processes. For example, the deep learning algorithm aims to mimic the activities of human neurons.

**Figure 6:**
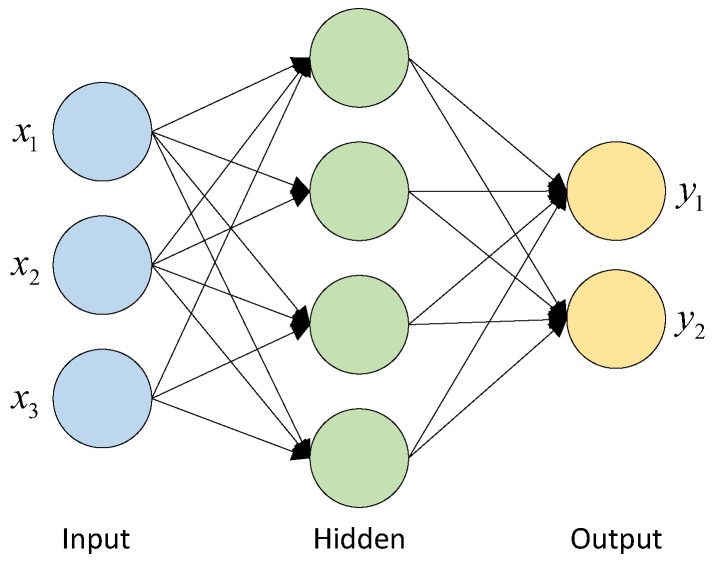
Basic structure of ANN.

The artificial neural network (ANN) has the features of massively parallel processing, distributed information storage, and good self-organizing and self-learning capabilities. As shown in Figure 6, ANN-based algorithms could be implemented by connecting numerous neurons with adjustable connection weights. They consist of an input layer, a hidden layer, and an output layer. The training samples could be the voltage measurements obtained from the numerical simulations.

The inputs to the neuron (input layer) usually contain one or more values (nodes), i.e., $x_{i}=\left\{x_{1},x_{2},x_{3},\cdots ,x_{n}\right\}$, while the outputs (output layer) can be expressed as $y_{i}=\left\{y_{1},y_{2},y_{3},\cdots ,y_{n}\right\}$. The hidden layer can be implemented with one or more layers. The number of nodes usually indicates the degree of non-linearity and robustness of the developed network.

**Figure 7:**
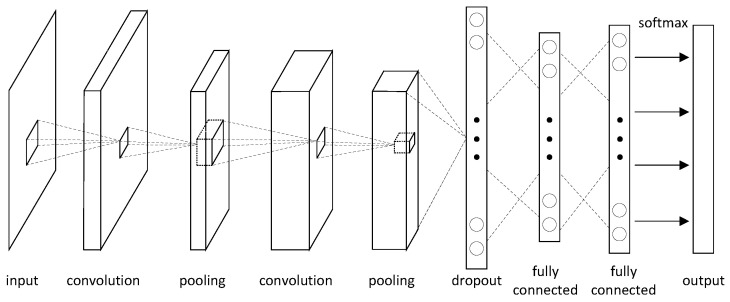
CNN structure designed for ERT image reconstruction.

Recently, the ANN algorithms are less discussed, and have been gradually replaced by the so-called deep learning algorithms, e.g., the convolutional neural network (CNN). Tan et al. has proposed a typical CNN structure, as shown in Figure 7 [57]. It has more neurons and more complex connections, i.e., the pooling layer, convolution layer, and dropout layer.

The performance of deep-learning-based image reconstruction algorithms usually relies heavily on the training process and the number of training samples. However, the training samples are usually difficult to collect. This may induce some difficulties in applying these intelligent algorithms in EIT image reconstruction. For example, the complicated shapes of lungs cannot be well reconstructed by using the circular inclusion training data, which is a common shortcoming of deep learning algorithms. To deal with this problem, Wei et al. developed a CNN-based method, which could reconstruct the details of investigated targets with sharp corners or edges [58]. Wu et al. improved the CNN-RBF method based on the radial basis function (RBF) [59]. Both of them adopt the pulmonary phantom model to test the performance of the proposed algorithm and verified that the proposed algorithm is capable of imaging the complex boundary of the lungs. Hamilton et al. demonstrated that more general networks could be trained without considering the boundary shape [54]. They proposed a novel algorithm that combines the CNN and D-bar algorithms, which could be useful in constrained imaging settings.

Zhang et al. proposed a deep CNN–V-shaped dense denoising net (VDD-Net) to improve the image quality [60]. The proposed method could preserve the sharp boundary features and correct the interference of the system. In addition, Li et al. used the algorithm of deep NN class and a pulmonary simulation model to solve the nonlinear problem of image reconstruction, even though the signal-to-noise ratio (SNR) was 30 dB [61]. This demonstrates the great potential of NN in removing the artifacts caused by system noise. Several works are displayed in Table 5.

It has been shown that the intelligent algorithms could find a large number of applications in the medical EIT, significantly extending the boundary of medical imaging technology. However, being a functional imaging modality, EIT technology still needs great work before it can be widely accepted. Firstly, AI systems are trained on large datasets, and if those datasets contain biased or unrepresentative information, the trained models can inherit and amplify those biases. Addressing data bias requires increasing diversity in training data and curating datasets carefully. In addition, overfitting occurs when a model becomes excessively tailored to the training data, which compromises the model’s ability to make accurate predictions on test data. To mitigate overfitting, techniques like regularization are commonly employed during the model training process. It should be noted that the researchers are developing techniques for improving data quality, reducing biases, and enhancing the robustness and generalization capabilities of AI models. By leveraging pre-existing knowledge, transfer learning or other methods could mitigate the limitations of small or biased datasets and reduce overfitting. In conclusion, further research is still needed to optimize the EIT image reconstruction algorithms and improve the interpretability of EIT images.

## 4. Applications

This section mainly focuses on the specific applications of EIT. First of all, the imaging of ventilation/perfusion (V/Q) is the main application scenario, which could offer useful information to indicate diseases. In addition, the diseases strongly related to EIT include acute respiratory distress syndrome (ARDS), pneumothorax (PTX), pulmonary embolism (PE), etc., which will be listed below. Some adjunct applications such as prone position or some novelty applications, e.g., lung transplantation or monitoring applied in the obese, will be introduced briefly at the end of this section.

### 4.1. Ventilation Detection

One of the most common biomedical applications in EIT is to monitor the ventilation distribution in patients treated in intensive care units (ICUs) [62]. The process of air moving in and out of the lungs is defined as ventilation [63]. Leonhardt and Lachmann conducted a series of experiments, showing that the variation in the end-expiratory lung impedance (EELI) correlates with that in the end-expiratory lung volume (EELV) during mechanical ventilation [21]. It has been proved that ΔEELI could be scaled and converted to volume (mL) by using the global tidal variation. The correlation between the preliminary volume and impedance constitutes the foundation of impedance-based ventilation detection.

The tidal volume (VT, or “TV” in some of the literature), is the gas volume inhaled during normal tidal breathing, which is a main parameter of ventilation. EIT could estimate the tidal volume with a high accuracy, due to the strong positive linear correlation that exists in the impedance variation ($\Delta Z_{\mathrm{breath}}$) and tidal volume. In addition, there is an agreement of the tidal volume measured from EIT (${\mathrm{VT}}_{\mathrm{EIT}}$) and spirometry (${\mathrm{VT}}_{\mathrm{SPIRO}}$) [64].

**Figure 8:**
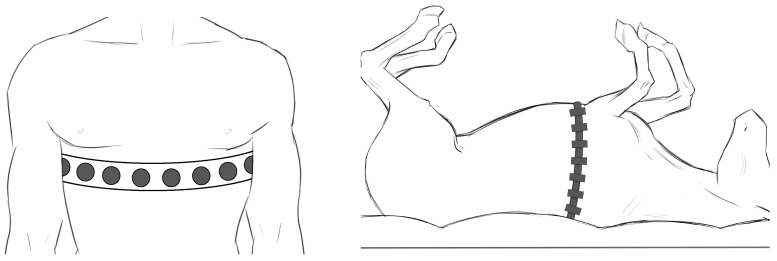
Schematic diagram of EIT electrode straps on humans (**left**) and horses (**right**).

Typically, EIT ventilation experiments will select animals such as horses, puppies of pigs or dogs, etc.; wherein the horse is the most common animal with a respiratory system of enormous functional plasticity [65]. Crivellari et al. extended the tidal volume detection from human to animal [63]. The difference is that the supine position is often employed in experiments on horses, while the standing position is more often used in human experiments, as shown in Figure 8.

In addition, Mosing et al. anaesthetized six horses and mechanically ventilated their lungs, which also obtained this positive correlation [66]. However, the linear relationship is still severely affected by the physical differences, so the best-fit lines ought to be employed to achieve an acceptable estimation.

EIT could monitor the ventilation distribution and provide dynamic functional images of lungs. In contrast, the image of CT is static and reflects the structural information. These two imaging techniques could cooperate to capture the ventilatory function while grasping structural lesions in lungs. In ventilation monitoring, the detection of tidal volume has been extended to the field of veterinary medicine, which has been accepted by the veterinary community [31]. In addition, ventilation monitoring can also coordinate with perfusion monitoring to indicate the disease caused by a mismatch between ventilation and perfusion, which will be introduced below.

### 4.2. Perfusion Detection

Pulmonary perfusion refers to the process of arterial blood delivered to the pulmonary capillary bed, which is equivalently as important as ventilation [67]. Recently, Safaee Fakhr et al. reported a severe dyspnoeic patient, and EIT was adopted to diagnose his pulmonary perfusion [68]. The patient has already been discharged from the hospital, and EIT has displayed the effectiveness in resolving perfusion defects.

**Figure 9:**
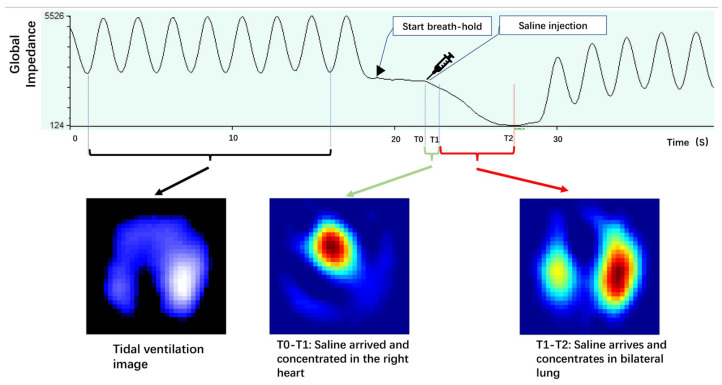
Impedance-time curves and corresponding ventilation/perfusion images during the saline bolus. Reprinted from [69].

The method frequently used in EIT is the conductivity contrast bolus (contrast-enhanced EIT or the saline bolus EIT method), which implies to inject a bolus of hypertonic saline (e.g., NaCl 10%) into the pulmonary circulation during a respiratory hold [69]. In this period, the co-action between the deficient tidal impedance and the relatively stable chest impedance makes the changeable impedance (caused by the saline bolus) indicate the forward lung blood flow. The bolus can leave a trace of impedance variation wherever it passes by, because the bolus conductivity differs from the blood conductivity [24]. Hence, the regions with greater impedance variation signify more saline passing through, and furthermore, higher perfusion exists. Figure 9 illustrates the impedance-time curve and the corresponding ventilation/perfusion images following the injection of saline bolus [69].

Frerichs et al. injected the hypertonic saline solution into three pigs, and generated the local time-impedance curves and functional-EIT (f-EIT) images, which verified the feasibility of f-EIT pulmonary perfusion imaging [70]. In addition, He et al. and Kircher et al. also used the hypertonic saline bolus in their articles [71,72]. He et al. proved that the EIT-based regional ventilation and perfusion were feasible to characterize the three main etiologies of acute respiratory failure [71]. However, the clinical applicability still needed more research. Kircher et al. presented an indicator-enhanced EIT-based pulmonary perfusion detection method [72], which could improve the robustness of spatial perfusion.

Pulmonary perfusion is evaluated mainly by the amplitude of regional EIT signal pulsatility [39]. However, the variation in amplitude of perfusion impedance is significantly smaller than the ventilation one [73]. Other differences could be found in Table 6. Kircher et al. added that EIT showed a great potential to track perfusion changes even in regional pulmonary compartments under complex pathological conditions [72]. Additionally, the mismatches could be taken into account in ventilation/perfusion distribution, as mentioned before.

### 4.3. V/Q Mismatch

Generally, moderate-to-severe cases of COVID-19 are accompanied by the physiological mismatch between alveolar ventilation (V) and pulmonary perfusion (Q) (V/Q mismatch), which is a hallmark derangement for ARDS patients, which may precipitate life-threatening consequences. Therefore, the in-time detection of V/Q mismatch is of vital importance. EIT can provide the regional distributions of V and Q, as well as their adequacy of matching [74,75]. Tingay et al. recorded the first case report of EIT to measure V-Q patterns, and found an important point according to which EIT recordings within the heart-rate domain were independent of heart movement [76]. The respiratory and heartbeat-related impendance-time signals could be separately given for the right lung, left lung, and anatomical heart region.

Thus, EIT is able to identify the small areas of tidal ventilation, which provides the possibility of dynamically measuring the V/Q mismatch. If defining three regions based on ventilation/perfusion patterns: regions only ventilated ($R_{V}$s), regions only perfused ($R_{P}$s), and regions that are both ventilated and perfused ($R_{V+P}$s), the matching level can be defined as follows:

(12) $${\mathrm{VQ}}_{Match\%}=R_{V+P}/\left(R_{V}+R_{P}+R_{V+P}\right)\times 100\%$$

Doan Trang Nguyen et al. proposed a wavelet denoising algorithm to separate perfusion signals from ventilation signals, so that the patient ventilation did not need to be interrupted [77]. Subsequently, the V/Q mismatch was assessed by the V/Q ratios of right lung to left lung, which could further detect PE. Spinelli et al. quantified the V/Q mismatch in a prospective observational study with 50 patients with different degrees of ARDS [78]. EIT measurements were still obtained by using a central venous bolus of saline (5%) during the end-inspiratory pause. The results conclude that EIT could evaluate the V/Q mismatch in the mechanically ventilated ARDS patients, as an efficient method to identify patients at higher risk of death and to guide individualized treatment. In the future, the quantification of V/Q mismatch will generate a huge push for the clinical application of EIT.

### 4.4. C-ARDS and NC-ARDS

ARDS is one of the most severe forms of lung disease [72], which has a high lethality. As a part of lung protective individualized ventilation strategy, EIT is an effective tool for guiding the mechanical ventilation and PEEP (positive end-expiratory pressure) titration [79,80]. For ARDS patients, choosing the most appropriate PEEP level is beneficial for opening collapsed alveoli or preventing excessive expansion of relatively normal/already opened collapsed lung areas. In addition, coronavirus disease 2019 (COVID-19) could result in a wide range of clinical manifestations, including ARDS [68,75]. The treatment of COVID-19 requires the consecutive monitoring of ventilation and inspiratory efforts [75], necessitating a harmless monitoring technology. Di Pierro et al. performed a clinical EIT-guided decremental PEEP trial and mechanically ventilated on patients, and demonstrated that EIT could be applied on the patients with either C-ARDS or NC-ARDS [81]. C-ARDS is the ARDS caused by COVID-19, while NC-ARDS is the ARDS aroused by other etiologies. These experiments build the foundation of using EIT to identify the C-ARDS and NC-ARDS.

The C-ARDS and NC-ARDS will lead to different specific characteristics in patients, which is the main principle to distinguish them. The C-ARDS exhibits a higher median best PEEP and a lower risk of overdistension at a higher PEEP level [81,82]. The obvious difference can also be seen in other characteristics such as respiratory mechanics and respiratory system compliance [82]. According to these physiological characteristics, EIT could diagnose and distinguish C- or NC-ARDS, and monitor the pulmonary condition of ARDS patients in the long term.

### 4.5. Pneumothorax

Pneumothorax (PTX) is a fatal disease in severe patients, the incidence of which is 8–10% in the ARDS patients [83]. EIT has been proven to be a feasible technique in detecting the PTX at the bedside [84,85].

Miedema et al. firstly described the events within the lung at the initiation of PTX, and demonstrated the distinct predictive changes in air-filling characteristics before the occurrence of PTX [86]. In addition, Yang et al. evaluated the routine use of EIT for the diagnosis of PTX in the ICU, and found that the ventral ventilation defect was a prominent feature in the early diagnosis of PTX in severe patients [87]. Girrbach et al. also demonstrated that EIT played an important role in the post-traumatic PTX with the reference of CT [88]. This finding will contribute to the combination of EIT and CT in future clinical PTX diagnosis.

### 4.6. Pulmonary Embolism

Pulmonary embolism (PE) is an acute condition blocking pulmonary perfusion and a common complication of COVID-19 [89]. Nguyen et al. predicted that the diagnosis of PE was the most probable application for EIT lung perfusion [73]. Subsequently, Nguyen et al. studied the contrast EIT of pulmonary perfusion defect caused by an artificially induced PE in a large ovine model (N = 8.78 ± 7.8 kg) [90]. Preliminary results showed that EIT could reliably detect the difference between normal and embolized lungs. Nguyen Minh et al. proposed an EIT-based bioimpedance solution reconstruction to reliably assess the severity of PE [89]. Recent research introduced that EIT was a potential diagnostic bedside tool in the diagnosis and follow-up of acute pulmonary embolism [91], which was verified by a series of case reports [92,93,94].

In addition, the hypertonic saline method could be applied to the testing of PE. Wang et al. presented a case report of high-risk PE, and the EIT monitoring with hypertonic saline bolus injection provided useful information for diagnosis and decision-making [95]. Prins et al. pointed out that the hypertonic saline bolus method was a promising tool to diagnose PE, and the usefulness ought to be determined in future research [91]. Table 6 shows the difference in several applications mentioned above.

### 4.7. Pulmonary Edema

Kunst et al. found the correlation between the EIT measurements and the amount of extravascular lung water (EVLW), which is an index of pulmonary edema [96]. It was concluded that EIT could reasonably estimate the amount of EVLW. Arad and Abboud verified this correlation by conducting an eight-electrode EIT experiment on humans. However, the quantified result has not been achieved [97].

Trepte et al. adopted a novel metric, i.e., the lung–water ratio, to reflect the total EVLW. The conclusions showed that EVLW could be determined reliably by assessing the characteristic changes observed on reconstructed images during lateral body rotation [98]. It gives an approach for the quantification of pulmonary edema, which has the potential to measure pulmonary edema at the bedside.

### 4.8. Prone Position

The prone positioning (PP) is one of the adjuncts for EIT clinical application. Perier et al. described the physiological effects of prone position on respiratory mechanics, ventilation, and perfusion. In their study, 41 patients with C-ARDS were recruited to collect the ventilated parameters in the supine position and prone position [99]. The result verified that the prone position improved the ventilation, and decreased the dorsal shunt and ventral dead space. However, the pulmonary compliance was not improved, probably due to the small sample size. Fossali et al. combined CT and EIT to investigate the lung recruitment and V/Q matching in patients with C-ARDS, similarly taking the prone position and supine position into account [100]. Consequently, the prone position had an impact on increasing recruitment, decreasing atelectrauma, and improving V/Q matching. Pierrakos et al. studied the global inhomogeneity and dorsal compliance of patients with COVID-19 before and after prone positioning [101]. The result demonstrated that the prone position improved the ventilatory homogeneity and dorsal compliance, and expanded the volume of the lungs. Subsequently, Wang et al. performed an EIT evaluation at the beginning, 3 h after the start, and at the end of prone position, which obtained similar results to the above [102].

These studies have shown that the prone position is a very effective tool in clinical practice to avoid measurement errors due to the influence of bad gestures. In addition, the prone position can improve the oxygenation of hypoxemic brain-dead donor’s lungs with atelectasis, which makes it feasible to improve the donor’s lung function and contributes to the lung transplantation [103].

### 4.9. Lung Transplantation

In 1994, Griffiths and Jossinet mentioned that the bioelectrical spectroscopy combined with multi-frequency EIT had a vital application in screening the viability of organs before the transplant surgery [104]. Edd and Rubinsky assessed the viability of 3D EIT in the field of organ transplantation [105]. Jiang et al. reviewed the use of ventilation- and perfusion-monitoring-based EIT in lung transplantation [106]. The research demonstrates that EIT has great potential in the transplantation field. Additionally, EIT may play a vital role in evaluating single-lung transplant patients before the transplant surgery, and make sense of the assessment of individual lung function during postoperative follow-up [107]. However, the author also pointed out that the current clinical information was not enough, which should be solved by further studies.

### 4.10. Pulmonary Monitoring in Obese Patients

Patients with obesity usually have some lung-related respiratory problems. EIT is one of the useful tools to systematically assess the respiratory mechanics and safely adjust for relatively high PEEP, which has been successfully used to evaluate the ventilation distribution to determine PEEP in obese patients [108]. When using the mechanical ventilation associated with esophageal manometry and EIT, the mortality of patients with respiratory failure and obesity was reduced by about 50% [109]. Fulton et al. also utilized EIT to measure ΔEELI as a primary outcome in high flow nasal oxygen therapy (${\mathrm{HFNO}}_{2}$) after bariatric surgery [110].

This application is very novel for EIT, but the main principle is the detection and monitoring of ventilation and perfusion, which has already been introduced in detail.

## 5. Conclusions and Perspectives

EIT has already been accepted as a promising tool for clinical imaging [111]. The greatest advantage is the high temporal resolution, as compared with other medical imaging modalities. It could provide dynamic clinical images and data at the bedside; therefore, it could be used for continuous lung imaging [65]. The use of EIT could achieve continuous breath-by-breath measurements, without the need to use a mask or intubation [79]. It has also been proved that EIT instruments are imperceptible and safe for the body, with no ionizing radiation exuded. Being a low-usage-cost and portable instrument, EIT could accommodate many clinical occasions. In Section 4, a number of clinical applications have been summarized and reviewed, and their possible applications have also been discussed.

However, there also exist several limitations, which may also directly affect the future development of EIT.

(1) Comparatively low spatial resolution. The EIT technique has been usually criticized for its low spatial resolution [65]. Great efforts have been undertaken to overcome this shortcoming, mainly by optimizing electrode array and image reconstruction algorithms. Despite these, the spatial resolution of EIT is still much lower than other clinical imaging modalities. This may stop EIT from being applied in the wide clinical fields. It could be concluded that it is almost common sense that the spatial limitation of EIT is about 5∼10% of the characteristic dimension of the phantom.

Due to its low spatial resolution, several ethical regulations ought to be taken into account. EIT should be used as a part of a comprehensive diagnostic approach rather than a standalone tool. Higher precision imaging tools should be involved to compensate for the shortcomings of EIT. In addition, patients and their families should be properly informed about the benefits, risks, and limitations of EIT.

(2) Shortcomings of 2D imaging. Currently, most clinical EIT instruments employ single-plane electrode arrays, allowing only slice imaging i.e., 2D imaging, rather than volumetric imaging. This configuration could only monitor one slice of the lungs at a time, while most CT scans can provide the images of entire lungs [69]. Although the EIT measurements carry 3D information on the electric field, it is difficult to achieve an accurate lesion location.

Therefore, it is an essential requirement to perform 3D imaging, which could monitor the entire lungs with dilated electrode belts. Three-dimensional imaging could provide an enhanced spatial resolution, which could potentially improve the diagnostic accuracy and avoid the shortcomings discussed before. In addition, it may also alleviate some problems that are frequently met in 2D imaging, e.g., the alignment of the electrode belt and the lesion location, for the 3D imaging region could comprehensively cover the lesion part.

Although the 3D EIT system is already available for laboratory use, there may still exist many difficulties while using it in a clinical environment. The achievement of high-quality 3D data could be a challenge, for more extensive electrode arrays with accurate positions are required, i.e., the computational complexity of image reconstruction will increase significantly, which brings great demands on efficient algorithms, data processing techniques, and high-speed hardware systems.

(3) Interpretability of EIT images. Unlike other “hard-field” tomography, EIT could only be viewed as a functional imaging modality. The information of EIT tomographic images is usually hard to be explained to a non-specialist, especially considering the low spatial resolution and conductivity map. Thus, more attention should be paid in developing the method to correlate the EIT images with some meaningful clinical parameters, which will play a vital role in identifying specific pulmonary conditions and expanding the application scope of EIT. However, these have still been seldom discussed in the previous literature and research.

In conclusion, EIT could be developed into a powerful bedside imaging tool for physicians to monitor pulmonary conditions and make treatment decisions. EIT could be a strong candidate for becoming a standard long-term care instrument and guiding personalized treatment. However, there is still a long way to go before EIT could be widely accepted in clinical applications.

All authors agree to the final version. Carefully check that the authors’ names and affiliations are correct, and that funding sources are correctly acknowledged. Incorrect author names or affiliations are picked up by indexing databases, such as the Web of Science or PubMed, and can be difficult to correct.

## Figures and Tables

**Table 1:** The comparison of electrode configuration with the placement.

Work	Electrode Array	Electrode Placement
Sophocleous et al. [17]	32-electrode textile belt	5–6th intercostal space
Hu et al. [29]	16-electrode textile belt	lower edge of breasts
Lin et al. [30]	32-electrode wireless belt	near the sixth rib
Zhao et al. [33]	16-electrode belt	5th intercostal space

**Table 2:** The comparison of time-difference algorithms.

Work	Algorithm	Result & Performance	Finding
Kang et al. [40]	A sub-domain based regularization method	IE (0.0444) CC (0.9392)	Regularization parameter with different weights performs better than constant weights.
Wang et al. [42]	A hybrid iterative optimization method	BR (0.1288) SSIM (0.9661) (without noise)	An efficient alternating minimization algorithm is introduced and image reconstruction quality in EIT is improved.
Wang [44]	A one-step proximal sparsity-promoting	RE (3.69) CC (0.9809) (Noise = 3%)	A reference approximation can be generated by NITR method as the starting value for the next sparsity-promoting step.
Sun et al. [45]	An I-TR method	RE (0.0786) CC (0.8972)	It can reflect the shape and the metastasis process of cancerous tissue more clearly.

**Table 3:** The comparison of time-difference and frequency-difference algorithms.

Algorithm	Advantage	Drawback
Time-difference	(1) Good real-time performance. (2) Sensitivity to high-frequency signals. (3) The algorithm is relatively simple.	(1) Relatively low resolution. (2) Affected by signal-to-noise ratio.
Frequency-difference	(1) High resolution. (2) Sensitivity to low-frequency signals.	(1) High algorithm complexity. (2) Poor real-time performance. (3) Sensitivity to high-frequency noise.

**Table 4:** The comparison of frequency-difference algorithms.

Work	Algorithm	Result & Performance	Finding
Hu et al. [46]	MWFD and EMWFD method	The detection of background obtains good performance.	When there are multiple backgrounds in the measured field, the imaging quality is relatively low.
Jiang and Soleimani [47]	An algorithm combining Tikhonov regularization and SIRT	Acceptable frequency-difference images can be obtained after calibration.	Background calibration requires both the background measurements and the anomaly measurements.
Cao et al. [48]	A proposed spectral constraints algorithm (SC)	Parameters (IN, SD, and PE) are reduced.	SC has stronger noise suppression and target identification abilities.
Bai et al. [49]	Combining td- and fd- method	Tackle with the scenarios where both bio- and non-conductive inclusions exist.	A wavelet-based fusion strategy is proposed to fuse the imaging results.

**Table 5:** The comparison of CNN-based algorithms.

Work	Algorithm	Result and Performance	Finding
Tan et al. [57]	CNN-based method	ICC (0.95) (without noise)	It has good generalization ability.
Wei et al. [58]	CNN-based inversion method (BE-SOM and DC-DLS)	Significant performance improvements while reconstructing targets with sharp corners or edges.	Be able to reconstruct triangular and rectangular inclusions and easily expand it to 3D.
Wu et al. [59]	Improved CNN method	RMSE (0.082) ICC (0.892)	It achieves high-resolution and robust shape reconstructions.
Zhang et al. [60]	VDD-Net	RE (0.140) SSIM (0.961)	Combining sensitivity theory and deep CNN model can better express the nonlinear relationship between the measurements and the parameters in the observation domain.

**Table 6:** Comparisons of the EIT applications.

Detection	Measurement Objective	Input Signal	Clinical Application
Ventilation	The opening and blockage of gas channels	low-frequency AC current	Ventilation management/respiratory monitoring/lung function assessment
Perfusion	The distribution of blood in pulmonary vessels	AC or pulse current sources of different frequencies	Pulmonary vascular function assessment/regional perfusion detection/interventions guidance
ARDS	The changes in V/Q patterns	AC current	Ventilation strategies guidance/lung recruitment assessment/progress monitoring of ARDS
PTX	The accumulation of air in the pleural space	low-frequency AC current	Diagnosis/monitoring/interventions guidance of PTX
PE	The regions of altered blood flow caused by PE	AC or pulse current sources of different frequencies	Pulmonary vascular function assessment/interventions guidance of PE
